# Validated Spectrophtometric Method for Simultaneous Determination of Buprenorphine and Naloxone in Pharmaceutical Dosage Forms

**Published:** 2017

**Authors:** Effat Souri, Farzaneh Sadat Ahmadi, Maliheh Barazandeh Tehrani, Majid Mohammad Hosseini, Sedigheh Fadaye Vatan

**Affiliations:** a *Department of Medicinal Chemistry, Faculty of Pharmacy and Drug Design and Development Research Center, Tehran University of Medical Sciences, Tehran, Iran.*; b *Department of Pharmaceutical Chemistry, Faculty of Pharmacy, Azad University, Tehran, Iran. *; c *Research and Development, Kish Medipharm Company, No. 64, Kish Free Zone Island, Iran.*

**Keywords:** Buprenorphine hydrochloride, Naloxone hydrochloride, Simultaneous, Derivative spectrophotometry

## Abstract

Buprenorphine is a partial mu agonist and kappa antagonist which is used for the treatment of pain and opioid addiction. A mixture of buprenorphine hydrochloride and naloxone hydrochloride has been approved for the treatment of opioid dependence.

In this study a third order derivative spectrophotometric method based on zero-crossing technique has been used for the simultaneous determination of buprenorphine hydrochloride and naloxone hydrochloride in tablets. The measurements were carried out at wavelengths of 257.8 (zero-crossing point of naloxone hydrochloride) and 252.2 nm (zero-crossing point of buprenorphice hydrochloride) for buprenorphine hydrochloride and naloxone hydrochloride, respectively in the third order derivative spectra obtained in methanol and 0.1 M NaOH (50:50) as solvent. The method was found to be linear in the range of 20-80 µg/mL for buprenorphine hydrochloride and 5-20 µg/mL for naloxone hydrochloride. The within-day and between-day coefficient of variation and error values were less than 2.5% and 1.8%, respectively. The proposed method was successfully used for simultaneous determination of these drugs in pharmaceutical dosage form without any interference from excipients or need to prior separation before analysis.

## Introduction

**Figure 1:**
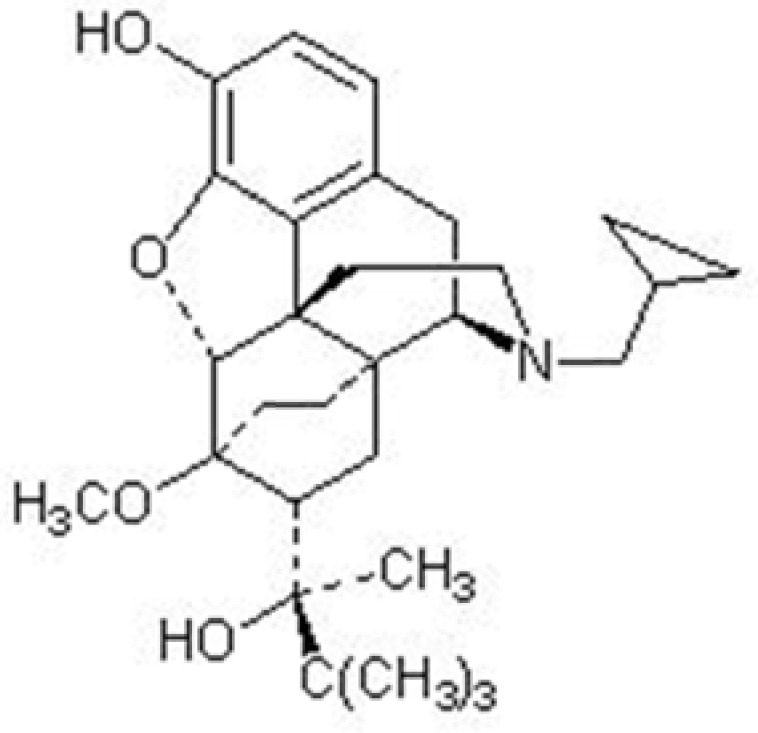
Chemical structure of buprenorphine

Buprenorphine, chemically known as 21-cyclopropyl-7α-[(S)-1-hydroxy-1, 2, 2-trimethylpropyl]-6,14-endo-ethano-6,7,8,14-tetrahydronororipavine (Figure 1.),is a semi-synthetic opioid. Buprenorphine is a partial mu receptor agonist and kappa receptor antagonist with analgesic activity without significant withdrawal symptoms (1).

**Figure 2:**
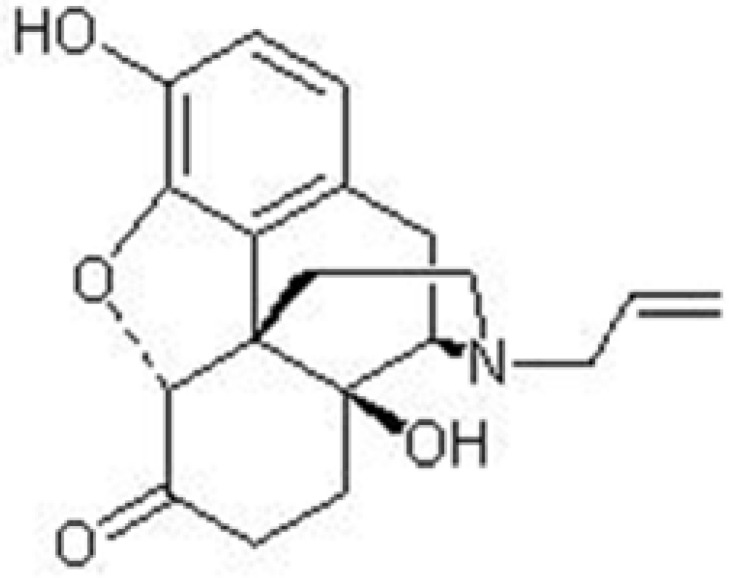
Chemical structure of naloxone

Naloxone, (5α)-4,5-epoxy,3,14-dihydroxy-17(2-propenyl) morphinan-6-one (Figure 2.), is a synthetic mu receptor antagonist. Naloxone is used for the treatment of opioid overdose and could induce the withdrawal syndrome in opiate-dependant individuals (1).

A combination of buprenorphine hydrochloride and naloxone hydrochloride is administered for the treatment of opiate dependence (2). Literature survey shows that several HPLC methods or LC-MS/MS methods are reported for the determination of buprenorphine alone or in combination with other drugs in biological fluids (3-9). There are also reports for the analysis of nalaxone in biological fluids using HPLC or LC-MS/MS (10-12). Determination of naloxone by HPLC in pharmaceutical dosage forms has also been reported before (13, 14) Buprenorphine and naloxone have been simultaneously determined in biological fluids using HPLC or LC-MS/MS methods (15-18). Few HPLC methods have been reported for simultaneous determination of buprenorphine hydrochloride and naloxone hydrochloride in dosage forms (19-21). Ion-pair formation and spectrophotometry has been used for the quantitive determination of bupernorphine hydrochloride alone in dosage forms (22).

To the best of our knowledge, no spectrophotometric method has been reported yet for simultaneous determination of these drugs in combination dosage forms. Most of the times, traditional spectrophotometric methods could not be used for the simultaneous analysis of multi-component mixtures because of their overlapping spectra. A mathematical derivative of the zero order absorbance spectra is an important development to solve this problem. In this study, derivative spectrophometry has been used for simultaneous determination of buprenorphine hydrochloride and naloxone hydrochloride. Spectrophotometric methods and zero-crossing derivative techniques have received increasing attention and great utility for determination of binary and multi-component samples with overlapping spectra (23-30). 

## Experimental

*Materials and methods*

*Materials*

Buprenophine hydrochloride was from Siegfried, Switzerland (Batch No: 1150L006) and naloxone hydrochloride was from Siegfried, Switzerland (Batch No: 1132L008). Both drugs were kindly provided by Kish Medipharm Pharmaceutical Company, Kish, Iran. Methanol and sodium hydroxide were of analytical grade and purchased from Merck (Darmstadt, Germany). The Buprenorphine/Naloxone 2/0.5 mg tablets (containing 2 mg buprenorphine hydrochloride and 0.5 mg naloxone hydrochloride) were from Kish Medipharm Pharmaceutical Company.

*Instrumentation*

Shimadzu UV-160A double beam spectrophotometer (Japan) with a fixed band width and 10 mm quartz cells were used for spectrophotometric measurements. The zero order and derivative spectra of working standard solutions of buprenorphine hydrochloride and naloxone hydrochloride were recorded over the wavelength range of 200-350 nm.

*Standard solutions*

Stock standard solutions of buprenorphine hydrochloride (1000 μg/mL) and naloxone hydrochloride (250 μg/mL) were prepared by dissolving appropriate amounts of bulk powder in a mixture of 0.1 M NaOH and methanol (50:50). Working standard solutions of buprenorphine hydrochloride (100 μg/mL) and naloxone hydrochloride (25 μg/mL) were prepared by subsequent dilution using the same solvent.

**Table 1:** Statistical data of calibration curves of buprenorphine hydrochloride and naloxone hydrochloride in mixtures with different concentrations using third order (   = 28.0) derivative spectra

**Parameters**	**Buprenorphine hydrochloride** a	**Naloxone hydrochloride** b
	^3^ **D**_257.8_ ** (Δλ = ** **28.0** **) **	^3^ **D**_252.2_ ** (Δλ = ** **28.0** **) **
**Linearity range**	20-80 (g/mL)	5-20 (g/mL)
**Regression equation**	Y =0.0174X+0.0359	Y =0.0211X+0.0554
**SD of slope**	0.00013	0.00025
**RSD of slope (%)**	0.76	1.17
**SD of intercept**	0.0004	0.0036
**Correlation coefficient**	0.997	0.997

a In the presence of naloxone hydrochloride (12.5 μg/mL)

b In the presence of buprenorphine hydrochloride (50 g/mL)

**Table 2:** Accuracy and precision data of determination of buprenorphine hydrochloride (20-80  g/mL) in the presence of naloxone hydrochloride (12.5  g/mL) by third order (   = 28.0) derivative spectrophotometry

**Concentration added** **(g**/**mL)**	**Concentration found** **(g/** **mL)**	**CV (%)**	**Error (%)**
Within-day (n = 3)			
20.00 50.00 80.00	19.97 0.25 49.95 0.16 79.71 0.81	1.27 0.33 1.02	-0.15 -0.10 -0.36
Between -day (n = 9)			
20.00 50.00 80.00	19.97 0.16 50.42 0.38 79.71 0.60	0.80 0.76 0.75	-0.15 0.84 -0.36

**Table 3:** Accuracy and precision data of determination of naloxone hydrochloride (5-20  g/mL) in the presence of buprenorphine hydrochloride (50  g/mL) by third order (   = 28.0) derivative spectrophotometry

**Concentration added** **(g/mL)**	**Concentration found** **(g/mL)**	**CV (%)**	**Error (%)**
Within-day (n = 3)			
5.00 12.50 20.00	4.91 0.09 12.47 0.15 20.06 0.15	1.76 1.18 0.75	-1.80 -0.24 0.30
Between -day (n = 9)			
5.00 12.50 20.00	4.99 0.11 12.47 0.11 20.08 0.17	2.27 0.86 0.84	-0.20 -0.24 0.40

**Table 4.** Comparison of the developed method with the reference method for the determination of tablets

**Compound**	**Label claimed(mg)**	**Found (mean ± sd)**		**Statistical Tests** *	
		**Proposed method**	**HPLC method**		
Buprenorphine HCl	2.00	2.01 ± 0.04	2.02 ± 0.01	t = 0.397	F = 0.063
Naloxone HCl	0.50	0.49 ± 0.03	0.49 ± 0.01	t = 0.184	F = 0.615

* Theoretical values of t and F at p = 0.05 are 4.303 and 19.00 respectively.

**Figure 3:**
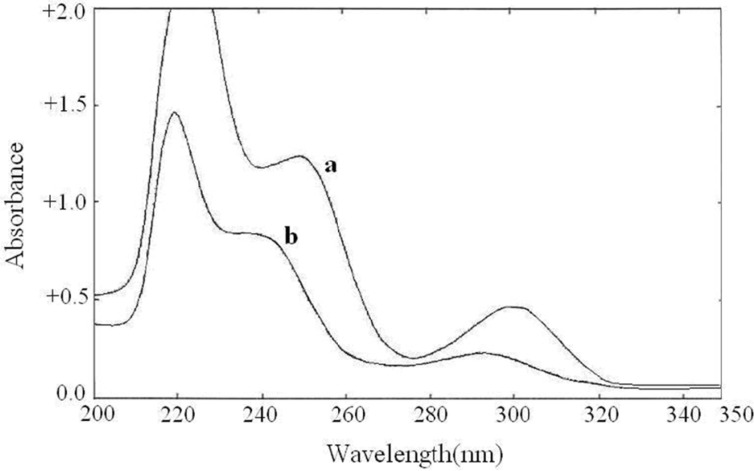
Zero order spectra of **(a)** buprenorphine hydrochloride (50  g/mL) and **(b)** naloxone hydrochloride (20  g/mL

**Figure 4:**
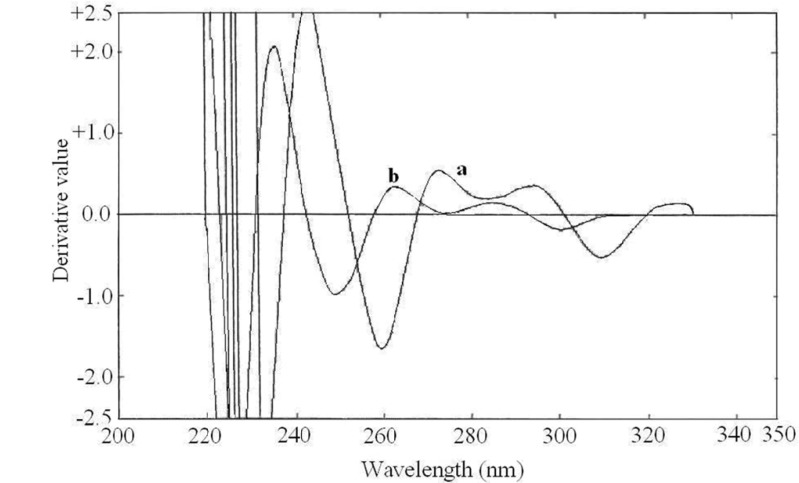
Third order derivative spectra of **(a)** buprenorphine hydrochloride (50  g/mL) and **(b)** naloxone hydrochloride (20  g/mL

*Derivative spectrophotometric method*

The spectrophotometric measurements were performed using the third derivative spectra (∆λ = 28.0) of buprenorphine hydrochloride and naloxone hydrochloride. The ^3^D amplitude at 257.8 nm (zero-crossing of naloxone) and 252.2 nm (zero-crossing of buprenorphine) were used for the determination of buprenorphine hydrochloride and naloxone hydrochloride, respectively.

*Linearity*

To find out the linearity of the spectrophotometric method, two sets of solutions were prepared. The first set was consisted of standard buprenorphine hydrochloride solutions with various concentrations (20, 30, 40, 50, 60, 70, and 80 µg/mL) in the presence of a fixed concentration of naloxone hydrochloride (12.5 µg/mL). The second set consisted of standard naloxone hydrochloride solutions at the concentration range of 5-20 µg/mL (5, 7.5, 10, 12.5, 15, 17.5, and 20 µg/mL) in the presence of fixed concentration of buprenorphine hydrochloride (50 µg/mL). Each solution was subjected to the spectrophotometric method and the calibration curves were constructed.

*Accuracy and precision*

The accuracy and precision of the method were evaluated by analyzing three sets of buprenorphine hydrochloride solutions at three different concentration levels (20, 50, and 80 μg/mL) in the presence of fixed concentration of naloxone hydrochloride (12.5 μg/mL). The same procedure was performed for naloxone hydrochloride solutions at 5, 12.5, and 20 µg/mL and fixed concentration of buprenorphine hydrochloride (50 µg/mL). The concentration of each solution was calculated using the corresponding calibration curve and the within-day accuracy and precision were calculated. The analysis was repeated for three consecutive days to find out the between-day accuracy and precision of the method.

*Application of the method in dosage form*

An average weight of finely powdered tablets (20 tablets), equivalent to one tablet, was transferred to a 10 mL volumetric flask. About 7 mL of a mixture of 0.1 M NaOH and methanol (50:50) were added and the mixture was sonicated for 20 min. Then, the solution was diluted to volume with the same solvent. After filtration through a syringe filter 0.45 µM (Teknokroma, Spain) and four times dilution, the resulted solution was subjected to the proposed spectrophotometric method. The data obtained was compared with a standard solution at the same concentration value to find out the amount of buprenorphine hydrochloride and naloxone hydrochloride in tablets. The tablets were also subjected to a previously reported HPLC method (19) to determine the amount of active compounds.

## Results and discussion

*Derivative spectrophotometric method*

The zero order absorption spectra of buprenorphine hydrochloride and naloxone hydrochloride, recorded against solvent blank, showed intensive overlap in the range of 200-350 nm (Figure 3.) which prevents the simultaneous determination of these drugs. Derivative spectrophotometric methods could be a useful solution for this problem. The first to fourth derivative spectra of the individual drugs were obtained in different ∆λ values. The spectrophotometric parameters such as the derivative order and ∆λ values were optimized to obtain the maximum sensitivity and reproducibility. The recorded spectra were examined to select suitable wavelengths in derivative spectra to be used for the simultaneous determination of buprenorphine hydrochloride and naloxone hydrochloride. At first methanol was used as the dilution solvent, but acceptable resolution and zero-crossing points were not observed. By using alkaline solvent, the derivative spectra pattern was changed and better zero-crossing points were obtained. As the drugs showed insufficient solubility in 0.1 M NaOH, a mixture of 0.1 M NaOH and methanol (50:50) were used as the dilution solvent. The zero-crossing points for both drugs in the third order derivative spectra (∆λ = 28.0) were assigned. Among several zero-crossing wavelengths the most suitable one was^ 3^D zero-crossing points of buprenorphine hydrochloride and naloxone hydrochloride at 252.2 nm and 257.8 nm, respectively (Figure 4.). At these points, the derivative value was proportional to the concentration of one of the drugs, while the derivative value of the other drug was near zero. The selected wavelengths showed acceptable sensitivity and reproducibility. The ^3^D value at 257.8 nm was proportional to buprenorphine hydrochloride concentration and not influenced by increasing the naloxone hydrochloride concentration. Also ^3^D value at 252.2 nm was dependent to the naloxone hydrochloride concentration.

*Linearity*

Using the synthetic solutions of varied concentrations of buprenorphine hydrochloride (20-80 μg/mL) in the presence of naloxone hydrochloride (12.5 μg/mL), the calibration curves were constructed over the concentration of buprenorphine hydrochloride. The results of six replicates are shown in Table 1. The same procedure was performed for synthetic solutions of naloxone hydrochloride (5-20 μg/mL) in the presence of buprenorphine hydrochloride (50 μg/mL) and statistical data were calculated which is shown in Table 1.

*Accuracy and precision*

The within-day and between-day accuracy and precision of the method were determined by analyzing three sets of each compound at three different concentration levels. The obtained results are shown in Tables 2 and 3. The within-day and between-day CV values for the determination of buprenorphine hydrochloride and naloxone hydrochloride were in the range of 0.3-1.3% and 0.8-1.8%, respectively, which is sufficient for assay quantification purposes.

*Application of the method*

Summary of the assay results of commercial tablets are shown in Table 4. which is in good agreement with the labeled amount. The results of this study were compared with a previously reported HPLC method by Student›s t-test and also F-test. The calculated t value and F value did not exceed the theoretical values, which indicated no significant difference between two methods. As the proposed method is much more simple, it could be used as a routine method for the simultaneous analysis of these drugs in pharmaceutical dosage forms in quality control laboratories.

*Relative recovery*

The relative recovery calculated by standard addition method, was 101.12 ± 0.59% and 100.76 ± 1.18% for buprenorphine hydrochloride and naloxone hydrochloride, respectively. These results revealed that there is on interferences from the tablet excipients and there is no need for pre-treatment of samples.

## Conclusion

Spectrophotometry could be a suitable alternative analytical method for time consuming and expensive HPLC methods. In this study, derivative spectrophotometric method has been used for simultaneous determination of buprenorphine hydrochloride and naloxone hydrochloride in tablets without the necessity of sample pre-treatment. 

The developed method is not only accurate, precise and simple, but also there is no need for time-consuming and expensive methods such as HPLC for simultaneous determination of these drugs. This method could be suggested for routine analysis of buprenorphine hydrochloride and naloxone hydrochloride for quality control purposes. 
